# Inflammation-related acute coronary thrombosis following vaccination: a case report

**DOI:** 10.1093/ehjcr/ytaf566

**Published:** 2025-11-05

**Authors:** Ayobami B Omodara, Jawahar Lal, Matthew D'Costa

**Affiliations:** Cardiology Department, Mid and South Essex Teaching Hospitals NHS Foundation Trust, Prittlewell Chase, Westcliff-on-Sea, Essex SS0 0RY, UK; Cardiology Department, Mid and South Essex Teaching Hospitals NHS Foundation Trust, Prittlewell Chase, Westcliff-on-Sea, Essex SS0 0RY, UK; Cardiology Department, Mid and South Essex Teaching Hospitals NHS Foundation Trust, Prittlewell Chase, Westcliff-on-Sea, Essex SS0 0RY, UK

**Keywords:** Acute total coronary occlusion, Myocardial infarction, Myocarditis, Vaccine-related myocarditis, Coronary angiogram, Cardiac magnetic resonance imaging, CRUSADE score, Case report

## Abstract

**Background:**

Acute chest pain and troponin changes in middle-aged individuals can pose a diagnostic challenge, as age-related vascular changes may complicate common inflammatory and infectious conditions, affecting outcomes. Nevertheless, research on vaccine-related myocardial injury and systemic inflammation causing acute coronary syndrome (ACS) remains limited when compared to the extensive literature and advances in ACS.

**Case summary:**

A 52-year-old man presented to A&E with a 2–3-day history of right-sided chest pain after receiving a flu vaccination. The pain radiated to his right shoulder and eventually to his central chest. He developed a fever after the vaccination and managed it with hydration and rest. He had a history of hypertension, dyslipidaemia, and ischaemic heart disease diagnosed by coronary angiogram in 2019. His electrocardiogram showed atrial fibrillation with a fast ventricular response, which was successfully chemically cardioverted. An angiogram revealed acute total thrombotic occlusion of the intermediate coronary artery branch. He underwent cardiac magnetic resonance imaging, improved, and was discharged with dual antiplatelet therapy and outpatient follow-up.

**Discussion:**

This case underscores the need to consider ACS in patients with high pretest probability of coronary heart disease, even with a strong suspicion of myocarditis. A thorough history, ECG, high-sensitivity troponin, echocardiography, and coronary angiography are key in assessing high-risk chest pain. Inflammatory heart conditions may independently increase the risk of acute plaque disruption and thrombotic occlusion leading to myocardial infarction.

Learning points

- We believe our patient likely acute coronary syndrome (ACS) secondary to severe systemic inflammatory response from the use of vaccine. Inflammatory heart conditions, including myocarditis and severe infections, may coexist with or trigger acute coronary plaque rupture and/or thrombotic occlusion.
- Acute coronary syndrome should be strongly considered in patients over 40 with high pretest probability of coronary disease, even with atypical symptoms (e.g. fever, paroxysmal atrial fibrillation (PAF), and flu-like symptoms). Coronary angiography is therefore essential when suspected to exclude treatable ACS.

## Introduction

Differentiating acute coronary syndrome (ACS) from other acute inflammatory cardiac conditions is particularly challenging in middle-aged patients, who often have coexisting atherosclerotic coronary artery disease (CAD). This case report highlights strategies for safely evaluating this population to avoid inappropriate therapy. Misdiagnosis can have serious consequences, including fatal ventricular arrhythmias, bleeding, and various forms of cardiomyopathy—both ischaemic and non-ischaemic; that significantly increase mortality.

## Summary figure

**Table:** Topic: Vaccine-related acute thrombotic coronary occlusion—a case report

Relevant timeline:	
Time	Event
October 2024 @ 00:00h	An ambulance arrives
	Initial ECG shows atrial fibrillation with fast ventricular response with ST depression in V1–V4. Discussed with CTC—likely rate-related changes
	Temperature 39.2°C
00:53	Aspirin 300 mg and i.v. paracetamol given
01:00	Arrived at accident C emergency
01:05	Assessment in resuscitation bay, ACE
01:19	ECG shows atrial fibrillation with fast ventricular response—i.v. metoprolol and magnesium administered
01:48	Preliminary differentials drawn up and treatment started, covering for myopericarditis/ACS/infective
	Endocarditis
02:54	ECG reverts to sinus rhythm with ventricular ectopic beats
03:03	Informed by laboratory of high troponin (904). Other notable results include raised
	CRP and WCC
03:05	Discussed with primary PCI centre due to ongoing chest pain and high troponin, advised for local management
03:05	Reviewed by the cardiology registrar
04:58	Admitted to the coronary care unit and underwent cardiac monitoring
Day 1	Echocardiogram reveals posterolateral wall motion abnormalities
Day 3	Invasive angiogram performed
Day 4	Discharged with DAPT for 1 year and then SAPT long term; high-dose statin, ACE inhibitor, and beta-blocker
Week 4	Cardiac MRI performed
Week 16	Patient followed up in outpatient clinic

DAPT, dual anti-platelet therapy; SAPT, single anti-platelet therapy (usually aspirin monotherapy).

## Case presentation

Our patient is a 52-year-old man with a background of hypertension and dyslipidaemia who was admitted to accident and emergency unit with a 2–3-day history of right-sided chest pain radiating to his right shoulder, which then gradually got more intense progressing to his central chest. All this started the day after receiving flu vaccination. He was later found to have high-grade fever (39.2°C) on the day of immunization and stayed at home for rest and hydration, as advised by his general practitioner (GP).

He has a history of CAD established on invasive coronary angiogram in 2017 and 2019. The most recent angiogram in 2019 showed mild atherosclerotic disease affecting both proximal left anterior descending atery (LAD) and circumflex arteries with moderate* disease in his intermediate branch artery (IR) but with good thrombolysis in myocardial infarction (TIMI) flow, angiographically. It was managed conservatively due to the location of the lesion (branch lesion) and chronic, stable atypical symptoms. He was subsequently lost to follow-up due to coronavirus disease (COVID)-19-related service disruptions in 2020.

He is an ex-smoker and non-alcoholic. He has never engaged in recreational drug use. He alluded to leading a sedentary lifestyle and had a body mass index (BMI) of 34 kg/m2.

On focused cardiovascular exam, he was tachycardic with irregular heart rhythm seen on the cardiac monitor as atrial fibrillation, rate 103/min. His jugular venous pulse (JVP) was normal, and his blood pressure was 126/74 mmHg. His precordial exam revealed no murmurs.

The chest appeared clear bilaterally, and he appeared euvolaemic. He also had rigour and chills and complained of generalized myalgia. His temperature was 39°C.

Relevant initial blood investigation showed a raised CRP of 203; high-sensitivity troponin (hsTroponin) I, 923; second hsTroponin I, 540; and C-reactive protein, 201 mg/dL (normal < 5). White cell (WCC) and neutrophil counts were raised. Haemoglobin (Hb), electrolytes, and renal function were all normal. D-Dimer was 228 ng/L (normal).

An initial electrocardiogram (ECG) taken by the ambulance crew showed sinus tachycardia with likely rate-related ischaemic features (global ST depression), which resolved spontaneously with normal heart rate. Electrocardiogram in A&E showed new onset atrial fibrillation with rapid ventricular rate, which cardioverted to sinus rhythm following i.v. metoprolol 5 mg within 30 min.

Chest X-ray was unremarkable, and a quick bedside echo done in A&E showed mildly reduced left ventricular (LV) function within the range of 50%–55% through visual estimation only. We concluded that this patient likely presented with post-vaccination myocarditis, but due to a high calculated GRACE** and HEART*** scores and relatively low bleeding score, we started on ACS protocol and prescribed full dose of anticoagulation with a CHA2DS2-VA**** score of only 1 and a bleeding score of zero.

Subsequent ECGs showed multiple ventricular and atrial ectopic beats, whilst his chest pain began to settle.

*Moderate disease in this context is defined as 50%–75% stenosis.
**Global registry for acute coronary events—GRACE
***History, Electrocardiogram, Age, Risk factors, Troponin—HEART
****CHA2DS2-VA score used to estimate the risk of stroke in atrial fibrillation and which stands for: C=Congestive heart failure (CHF), H=Hypertension, A=Age 65–74 scores 1, Age 75 and above scores 2; D: Diabetes mellitus: 1, Stroke/TIA/hromboembolism: 2; Va: Vascular disease (prior MI, PAD, Aortic plaque: 1).

He was then admitted to the coronary care unit and had an angiogram the next day. See illustration below:

An inpatient angiogram performed the next day showed an acute total occlusion of the intermediate artery with a TIMI flow of 0 (red circle above). There was residual stable mild disease in the proximal LAD, the same as was found on the previous angiogram.

According to the interventional team, this patient most likely had the artery infarcted 2–3 days earlier, and the LV angiogram revealed anterolateral wall motion hypokinesia but good overall function.

The diagnosis was therefore acute thrombotic occlusion (ATO) of the intermediate coronary artery, currently symptom free. The recommended treatment strategy was a conservative approach with dual anti-platelet treatment (DAPT) with aspirin and clopidogrel for 6 months and then lifelong single antiplatelet treatment (SAPT).

Following a patient-centred approach and shared decision-making process, the patient opted to delay commencement of an anticoagulant. He continued to have his beta-blocker and high-dose lipid-lowering therapy—atorvastatin 80 mg every night.

**Figure 1:**
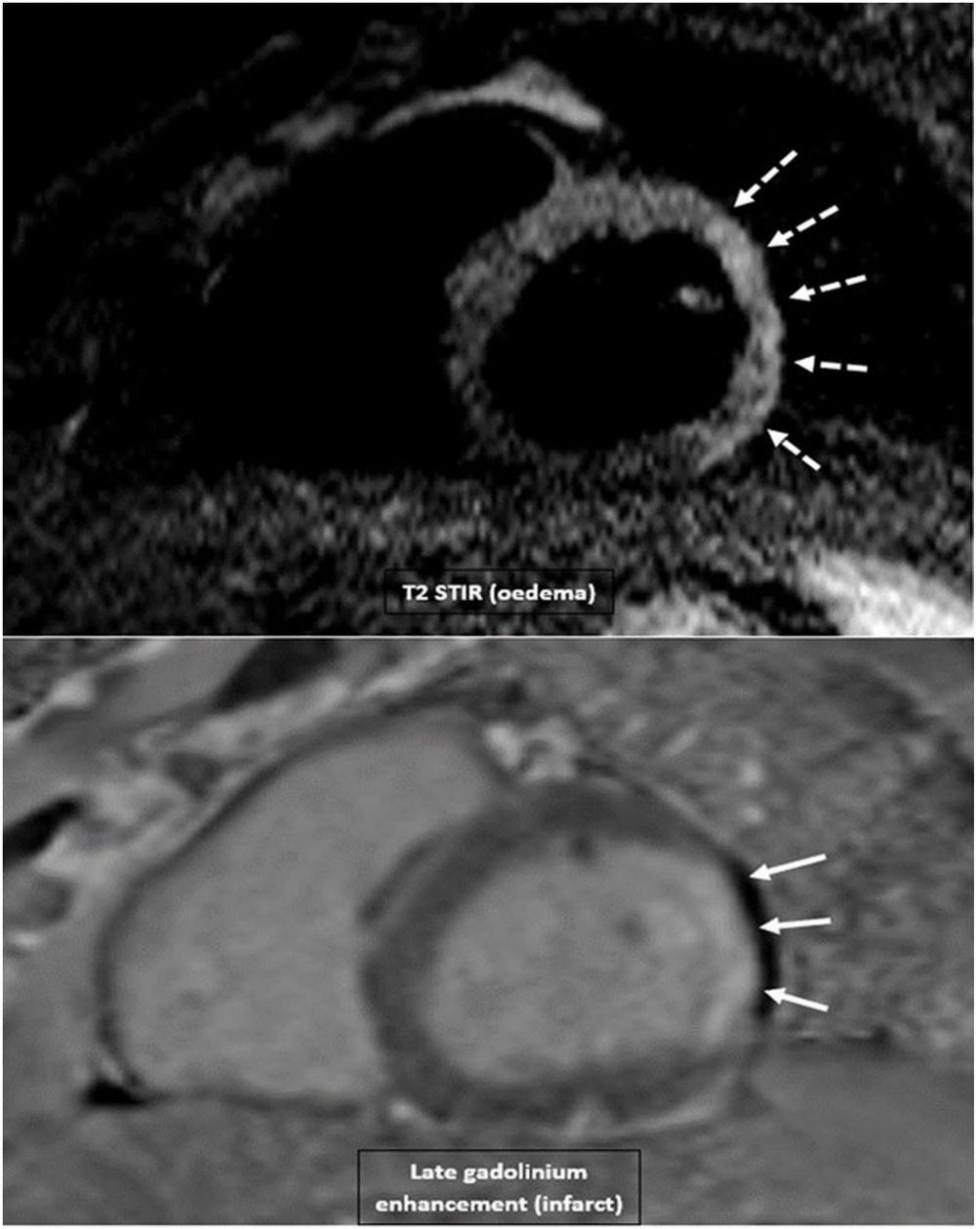
The broken arrows depict: myocardial oedema; solid arrows shows: infarct late gadolinium enhancement (LGE).

To rule out co-existent myocarditis, which in his case would be of long-term prognostic significance, urgent cardiac magnetic resonance imaging (CMRI) was recommended within the next 4 weeks. See *Figure 1*

Cardiac magnetic resonance rest protocol confirmed regional wall motion abnormality and territorial myocardial oedema on T2-weighted short tau inversion recovery (T2 STIR) (white broken arrows). It also shows late gadolinium enhancement (LGE), which points to the infarcted area of the myocardium (white solid arrow). These findings are consistent with ACS. His LV ejection fraction was 50%.

## Discussion

Upon thorough review of the patient’s history and completion of a full investigation, it is now clear in retrospect that he had occlusive ACS. He had a previous elective computed tomography coronary angiogram (CTCA) due to atypical symptoms and subsequently an invasive angiogram confirming severe proximal intermediate ramus (IR) stenosis. This was left without intervention as it was a proximal branch lesion that was not angiographically or clinically significant.

Additionally, this case highlights the common challenge of ruling out co-existent inflammatory cardiac disease during the early stages of suspected acute coronary events. Given these considerations, we decided to discuss myocarditis as a possible key differential diagnosis together with the effects of systemic inflammatory response on triggering ACS and outline the steps we took to exclude it.

Acute myocarditis can also mimic ACS as a cause of non-ischaemic myocardial injury. It is often underdiagnosed due to non-specific symptoms such as chest pain, elevated troponin, and abnormal ECG.^1^ In high-risk patients, severe systemic inflammatory process may also trigger occlusive ACS. Delayed contrast-enhanced cardiac MRI (CMR) is the gold standard for distinguishing cardiac inflammatory disease from myocardial infarction.^1,2^

Viral infections are the leading cause of myocarditis.^3^ Other causes include vaccine-induced, toxic (e.g. chemotherapy, marijuana, and cocaine), drug-induced hypersensitivity, and hyper-eosinophilic syndromes.^4^ Vaccine-induced cardiac inflammation has been documented in several studies, including in an observational study by Barton *et al*.,^4^ which described acute and subacute hypersensitivity reactions to certain vaccines, such as smallpox and COVID-19, resulting in eosinophilic myocarditis.^4,5^

The presence of flu-like symptoms and high temperature added to the diagnostic uncertainty, especially without classic ischaemic electrocardiographic (ECG) changes. The link between viral influenza infection and/or vaccination and inflammatory cardiac disease leading to an acute coronary event remains poorly studied and weakly established in medical literature. A review by Baral *et al.*^6^ attempted to explore this correlation but found a very low incidence of, and complications from, influenza-related myocarditis. Moreover, viral infections and vaccinations, such as COVID-19, have been associated with cardiac inflammation and acute coronary artery occlusions due to platelet dysregulation and endothelial dysfunction, as highlighted in several recent studies.^7,8^ We suspect that a comparable mechanism may have contributed to this gentleman’s acute coronary event.

**Figure 2:**
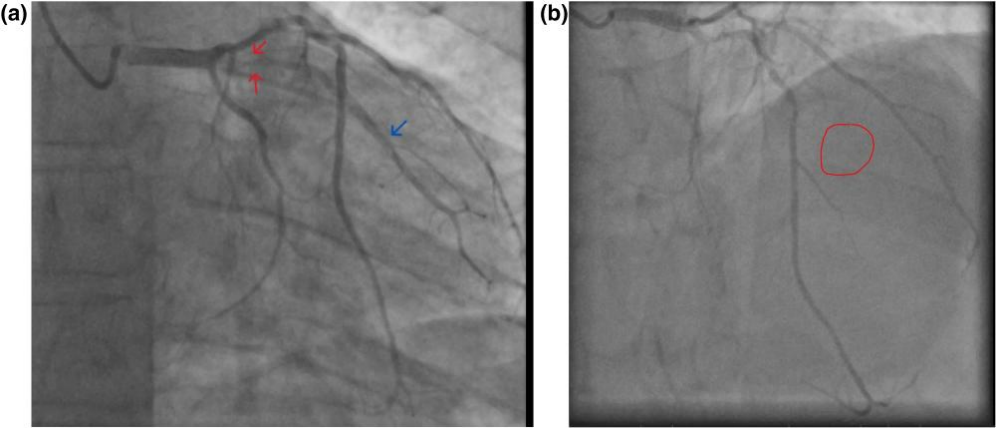
(*A*) Invasive coronary angiogram in 2019. Red arrows show severe proximal intermediate branch stenosis according to this coronary angiogram in 2019. The blue arrow shows the intermediate artery branch, which was no longer visible on the angiogram from 2024. (*B*) Acute thrombotic occlusion of the intermediate artery. Red circle marks the disappearance of flow down the intermediate artery.

According to the European Society of Cardiology (ESC), guidelines for treating individuals presenting with chest pain who are at risk for ischaemic heart disease (IHD) strongly recommended coronary angiography to rule out acute coronary events.^9,10^ The findings, according to *Figure 2*, raised a question around the potential for severe subacute and acute illness as a precipitant of a major coronary event in the form of ACS.

However, it is not uncommon for individuals to be diagnosed with both severe infection and/or an inflammatory process and acute Type 1 (thrombotic) myocardial infarction simultaneously. Several theories have been proposed, including plaque instability and rupture due to inflammatory changes, demand ischaemia, and reduced systolic function, which can disrupt homeostasis and lead to thrombosis and myocardial infarction (MI) in certain individuals.^11^

It is therefore imperative to perform a coronary angiogram in all suspected cases of inflammatory processes relating to the heart such as myocarditis. This is because patients with true myocarditis are high risk of ventricular arrhythmias (VAs) and sudden cardiac death (SCD) especially in the acute phase. Additionally, ∼10% of confirmed cases progress to dilated cardiomyopathy and/or severe heart failure.^12^

One useful tool to differentiate between ACS and myocarditis is the SAMY score, which considers factors such as C-reactive protein levels, age, leucocyte count, timing of infection, dyslipidaemia, and hypertension as well as Lake Louise criteria in CMRI.^13^

Despite a comprehensive 2023 ESC guideline statement,^13^ a significant research gap remains differentiating between acute MI and myocarditis, particularly in resource-limited hospitals far from percutaneous coronary intervention (PCI) centres. Emerging studies on microRNA show promising results, and CMR, with or without endomyocardial biopsy, remains the gold standard.^7^

Our patient was reviewed in the cardiology clinic after 3 months and had made complete clinical recovery with excellent medication adherence. Following AF-CARE***** pathway as recommended by ESC, this patient, given his age and how physically active he was, opted to only address his risk factors with aggressive lifestyle modification and secondary prevention and continued to lose weight. He agreed to follow-up in 6 months’ time for further review and re-discussion. *****AF-CARE stands for: AF: Atrial fibrillation; C: Comorbidity and risk factor mnagement; A: Avoidance of stroke and thromboembolism; R: Reduce symptoms by rate or rhythm control; E: Evaluation and dynamic reassessment. See the European Society of Cardiology website for further reading.

## Data Availability

The data underlying this article are available in the article and in its online supplementary material.

## Lead author biography

Dr Ayobami Benedict Omodara is currently in his second year of Cardiology residency (ST5) in the England, UK. He earned his primary medical qualification from Lviv National Medical University, Ukraine, graduating in the top 10% of his class. His primary interests lie in structural heart disease, structural and coronary interventions, arrhythmogenic heart conditions, sports and preventive cardiology. Additionally, he is an academic enthusiast and actively engages in academic writing and teaching.

## Notes

**Consent:** The patient(s) described in this manuscript has/have given written informed consent to the publication of the case details, including any accompanying images. The authors confirm that this consent has been obtained in accordance with the ethical standards outlined by the Committee on Publication Ethics (COPE). All reasonable efforts have been made to ensure patient anonymity; however, complete anonymity cannot be guaranteed.
